# Endoscopic double-layer nylon rope ligation for rescue of gastric ulcer bleeding after failed OTSC treatment: A case report (with video)

**DOI:** 10.1097/MD.0000000000050817

**Published:** 2026-10-02

**Authors:** Dan Wu, Hong Wei, Da Yong Sun, Xiang Yu Wang

**Affiliations:** a Department of Gastroenterology, The Second People’s Hospital of Shenzhen City (The First Affiliated Hospital of Shenzhen University), Shenzhen, Guangdong, China.

**Keywords:** case report, endoscopic treatment, gastric ulcer bleeding, nylon rope ligation, OTSC, rescue therapy

## Abstract

**Rationale:**

The over-the-scope clip (OTSC) system is a potent endoscopic hemostatic tool. Endoscopic nylon rope ligation, a mature, minimally invasive technique for treating colonic polyps, has rarely been reported for gastrointestinal bleeding or as a rescue therapy after OTSC failure. The use of endoscopic double-layer nylon rope ligation as a rescue treatment for peptic ulcer bleeding following OTSC failure in this case is the first such report.

**Patient concerns:**

A 61-year-old man was admitted to the emergency department with “hematemesis and melena for 3 days,” accompanied by dizziness and fatigue.

**Diagnoses:**

Bleeding gastric ulcer (Forrest IIa type).

**Interventions:**

Hemostatic clip application for the vascular stump at the center of the ulcer, submucosal injection-OTSC–double-layer nylon rope ligation.

**Outcomes:**

Immediate hemostasis was achieved after double-layer nylon rope ligation, with no recurrence of hematemesis or melena. The patient’s vital signs stabilized, and hemoglobin concentration increased to 72 g/L at 24 hours postoperatively. Gastroscopic reexamination at 2 weeks revealed shedding of the OTSC and nylon ropes, with a clean ulcer surface. At 8 weeks after endoscopic treatment, the ulcer presented new red mucosa with slight mucosal convergence. At the 4-month follow-up, the patient was asymptomatic, and the ulcer had completely healed.

**Lessons:**

Double-layer nylon rope ligation is a safe, effective, and easy-to-perform rescue therapy for gastric ulcer bleeding after OTSC failure.The core advantages of this method include enhanced compression stability, minimal tissue damage, and high operational efficiency. It is particularly suitable for bleeding scenarios with endoscopically accessible lesions, large-caliber blood vessels, or significant tissue edema, providing a new clinical option for such high-risk bleeding cases.

## 1. Introduction

Acute upper gastrointestinal (GI) bleeding is a common and potentially life-threatening condition, with nonvariceal bleeding accounting for 80 to 90% of its etiology. Gastroduodenal peptic ulcer is the most common primary cause.^[1,2]^ Internationally recognized treatments for peptic ulcer-related bleeding include acid suppression and conventional endoscopic therapies, such as spraying, electrocoagulation, and conventional hemostatic clip application.^[3–5]^ However, after these treatments are performed, approximately 8 to 15% of patients still experience persistent or recurrent bleeding, and the mortality rate of peptic ulcer bleeding patients remains between 10 to 14%.^[6,7]^ The American College of Gastroenterology released an updated nonvariceal upper GI bleeding guideline in 2021, which recommends that for some nonvariceal upper GI bleeding patients, if conventional electrocoagulation and hemostatic clip therapy fail or are predicted to be ineffective, over-the-scope clip (OTSC)s should be considered for hemostasis.^[8]^ Nevertheless, this association suggests that even now, certain clinical situations may justify the use of OTSCs as first-line therapy for patients with peptic ulcers with bleeding,^[6,7]^ including those with large ulcer beds with visible blood vessels that may not be suitable for the use of standard mechanical or thermal therapies.^[9,10]^

Compared with traditional endoscopic clips, the OTSC system is made of contractible superelastic nitinol, providing better tissue adherence. Studies have shown that the clinical success rate of OTSCs is 80 to 90%, indicating that even with the use of OTSCs, a 10 to 20% risk of rebleeding remains.^[11,12]^ Most of these rebleeding patients have large, fibrotic ulcers. When the mucosa surrounding the ulcer is severely edematous, the tissue is fragile, or when the anatomical location is unique, OTSCs are prone to incomplete clipping and fixation difficulties, leading to hemostatic failure. At this time, prompt effective rescue measures must be taken.^[13]^ Surgical and interventional radiology are conventional rescue treatments,^[14,15]^ however, they may fail to achieve hemostasis and are associated with more complications. A United States study revealed that for patients with recurrent GI bleeding after endoscopic therapy, the cost and reimbursement amount are greater when angiography or surgery is used as rescue treatment.^[16]^ Reports regarding rescue methods for patients with gastric ulcer bleeding and rebleeding after OTSC are scarce, and the reported methods often involve further surgical intervention. Whether other hemostatic methods are possible requires further exploration. Endoscopic resection of colonic polyps after nylon rope ligation is a mature, minimally invasive technique, and its clinical application and safety have been verified in numerous studies.^[17]^ However, it has rarely been reported for GI bleeding, and even more so as a rescue therapy after OTSC failure. This study reports the case of a patient with gastric ulcer bleeding for whom OTSC failed and for whom hemostasis was successfully achieved with double-layer nylon rope ligation. The patient was followed for 4 months, and the ulcer healed well, suggesting that this method is safe and effective as a rescue treatment for gastric ulcer bleeding after OTSC failure. The application value and key operational points are discussed in combination with a review of the literature to provide a reference for clinical practice.

## 2. Case presentation

This study was conducted in accordance with the Declaration of Helsinki and was approved by the Ethics Committee of the Second People’s Hospital of Shenzhen City (First Affiliated Hospital of Shenzhen University) (Ethics Approval Number: 2025-733-01PJ). Written informed consent was obtained from the patient for publication of this case report and accompanying images/video.

A 61-year-old man was admitted to the emergency department on March 5, 2020, with “hematemesis and melena for 3 days” accompanied by dizziness and fatigue. He had a medical history of hepatitis B cirrhosis, which was well controlled with long-term antiviral medication, and hypertension, which was also well controlled. He underwent cholecystectomy for gallstones more than 10 years prior, with good postoperative recovery, and minimally invasive surgery for mid-ureteral calculi more than 30 years prior, with details unknown. He denied a history of diabetes, coronary heart disease, long-term use of anticoagulant or antiplatelet drugs, and other surgical or traumatic conditions.

### 2.1. Physical examination

The patient’s temperature was 36.8°C, pulse was 108 beats/minute, respiratory rate was 22 breaths/minute, and blood pressure was 96/56 mm Hg. The patient was conscious but lethargic, with pale skin and nail beds. The abdomen was soft, with mild tenderness in the upper abdomen and no rebound tenderness or muscle guarding. The liver and spleen were not palpable below the costal margins. Shifting dullness was negative, and bowel sounds were active (8 times/minute). No edema was noted in the lower extremities.

### 2.2. Auxiliary examinations

Laboratory tests revealed a hemoglobin concentration of 68 g/L (normal reference range: 120–160 g/L), a hematocrit of 20%, a white blood cell count of 10.5 × 10^9^/L, and a platelet count of 156 × 10^9^/L. Coagulation function tests revealed a prothrombin time of 12.5 seconds, an activated partial thromboplastin time of 35.2 seconds, and a fibrinogen concentration of 2.8 g/L. Liver function, kidney function, electrolyte levels, blood glucose levels, carcinoembryonic antigen levels, carbohydrate antigen 19-9 levels, and alpha-fetoprotein levels were within normal limits.

Emergency gastroscopy (GIF-H290; Olympus, Tokyo, Japan): No abnormalities were found in the esophagus or duodenum. An irregular ulcer measuring approximately 0.8 × 1.0 cm was observed in the middle-upper segment of the greater curvature of the gastric body. The base of the ulcer was covered with yellowish-white exudate, with a suspected vascular stump, and no active bleeding was noted. Significant congestion, edema, and erosion were noted in the mucosa surrounding the ulcer.

### 2.3. Treatment process

#### 2.3.1. Conventional endoscopic therapy

During emergency gastroscopy, a suspected Forrest IIa ulcer was found on the greater curvature of the gastric body (Fig. 1). Hemostatic titanium clips were applied to occlude the vascular stump, and 1:10000 epinephrine was injected submucosally around the ulcer. No obvious bleeding was observed after the procedure, and the patient returned to the ward safely. Further symptomatic treatments, including fasting, acid suppression, fluid replacement, and blood transfusion, were continued. On the 3rd postoperative day, the patient developed melena again (black watery stool, approximately 300 g), accompanied by mild dizziness. Blood pressure was 90/60 mm Hg, heart rate was 92 beats/minute, and hemoglobin concentration was 65 g/L (previously increased to 73 g/L after blood transfusion). Active bleeding could not be excluded, and a repeat gastroscopy was performed.

**Figure 1.**
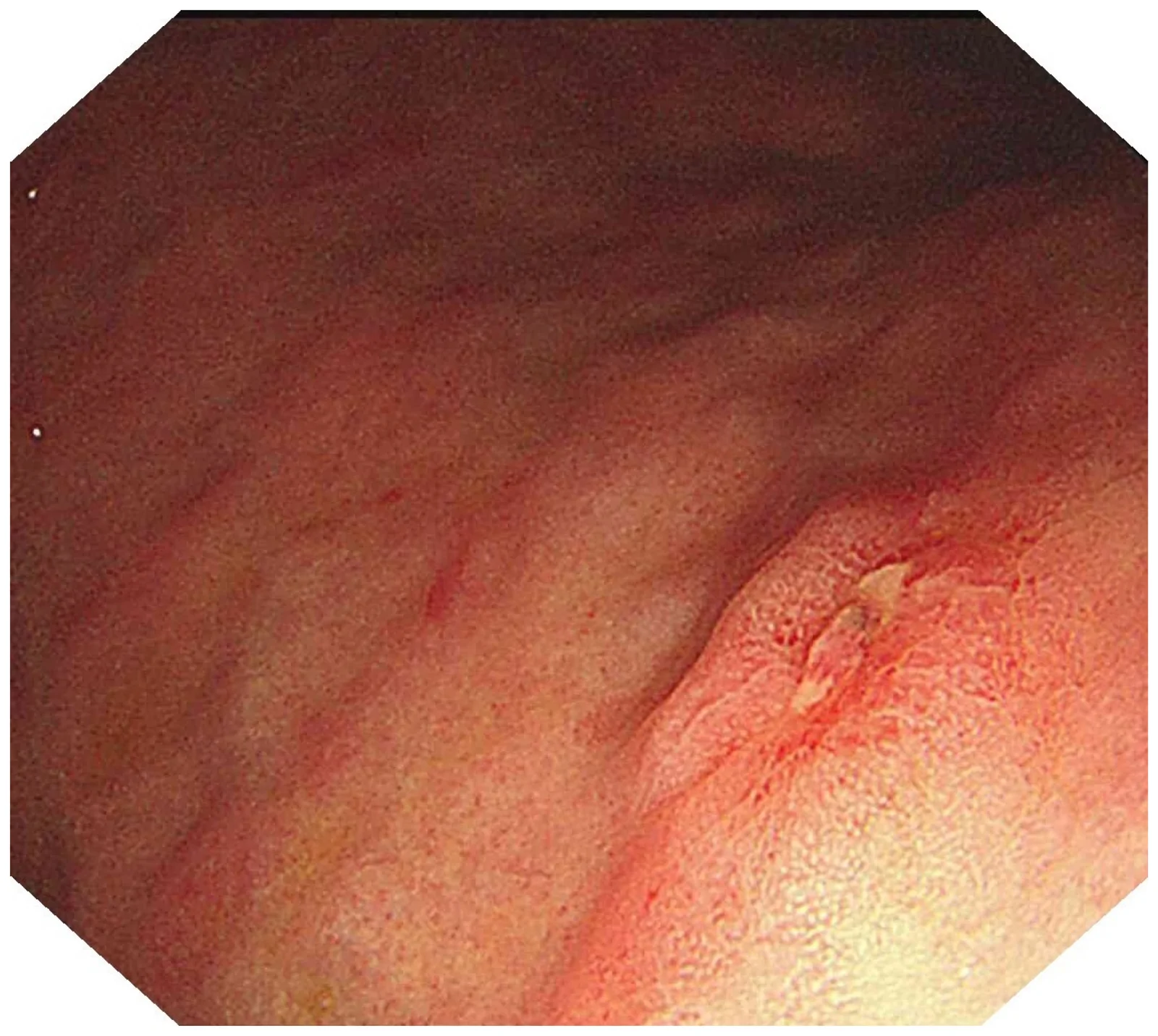
An irregular ulcer measuring approximately 0.8 cm × 1.0 cm was observed in the middle-upper segment of the greater curvature of the gastric body. The base of the ulcer was covered with yellowish-white exudate, with a suspected vascular stump, and no active bleeding was noted.

#### 2.3.2. OTSC hemostatic clipping

Gastroscopy was performed on the 4th day after conventional endoscopic hemostasis. A small amount of dark red blood residue was found in the gastric cavity, and the hemostatic clips at the ulcer site had fallen off. The base of the ulcer was covered with yellowish-white exudate, and a vascular stump approximately 0.3 cm in diameter was clearly visible in the center (Forrest IIa type) (Fig. 2A and 2B), with no active bleeding. Significant congestion, edema, and erosion were noted in the mucosa surrounding the ulcer. Considering the poor efficacy of conventional endoscopic hemostasis, OTSC treatment was selected. An OTSC (model HCCD-0-195-M-C; Micro-Tech, Nanjing, China) was mounted on the distal tip of the endoscope via a preloaded transparent cap and advanced to the lesion. The ulcer base, including the visible vascular stump and surrounding edematous mucosa, was suctioned into the cap under direct endoscopic vision. Once adequate tissue acquisition was confirmed, the clip was deployed to grasp and compress the ulcer and surrounding tissues. During the procedure, the tissues around the clip were well occluded with no active bleeding, and the operation was successful (Fig. 2C). The patient returned to the ward safely, and fasting, acid suppression, blood transfusion, and fluid replacement were continued. On the 2nd day after this procedure, the patient developed hematochezia again (dark red blood sample, approximately 400 g), accompanied by sweating, dizziness, and palpitations. His pulse was 112 beats/minute, respiratory rate was 20 breaths/minute, and blood pressure was 88/54 mm Hg. Active bleeding was considered, and immediate symptomatic treatments, including fluid replacement, volume expansion, blood transfusion, and antishock therapy, were administered, followed by emergency gastroscopy.

**Figure 2.**
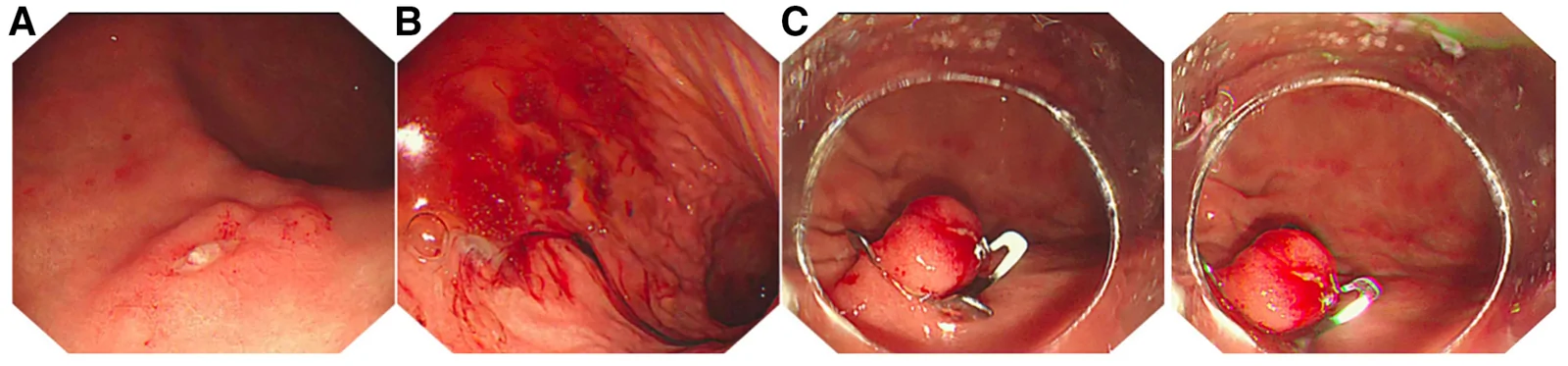
(A) The ulcer had increased in size compared with the previous examination. The base of the ulcer was covered with yellowish-white exudate, and a vascular stump approximately 0.3 cm in diameter was clearly visible in the center, with no active bleeding. (B) A small amount of dark red blood residue was found in the gastric cavity. (C) The ulcer was treated with an over-the-scope clip, and the procedure was successful. (D) After OTSC therapy, the ulcer crater shrank, with no obvious vascular stump visible. OTSC = over-the-scope clip.

#### 2.3.3. Rescue therapy (double-layer nylon rope ligation)

Operational steps: First, the endoscope was reinserted into the gastric cavity, where a large amount of dark red blood residue and blood clots were found. After repeated irrigation, intermittent jet bleeding was observed at the OTSC site. The lesion was repeatedly irrigated with normal saline, and the OTSC clip was in place, clearly exposing the location of the vascular stump (Fig. 3A and 3B). The therapeutic endoscope (GIF-Q260J; Olympus, Tokyo, Japan) was replaced and equipped with an endoscopic nylon rope ligator (LeoMed, Loop20-LD230). This device is a single-layer ligator that delivers 1 nylon rope at a time. The nylon rope is loaded onto the ligator, advanced through the endoscopic working channel to the target site, and released to form a ligature ring. Normal saline and ice-cold noradrenaline saline (8 mg/100 mL) were routinely prepared. Second, first-layer ligation: a nylon rope was loaded onto the ligator and inserted into the vicinity of the ulcer bleeding site through the endoscopic working channel. At the root of the OTSC, nylon rope was deployed to complete the first-layer ligation, and the ligature ring was observed to tightly compress the surrounding tissues, resulting in a significant reduction in bleeding (Fig. 3C). Although bleeding was significantly reduced after the first ligation, considering the relatively large caliber of the vessel (approximately 0.3 cm), the severe edema and fragility of the surrounding mucosa, and the strong peristaltic activity of the gastric body’s greater curvature, we were concerned that a single ligature might be prone to slippage under mechanical traction. Therefore, to enhance hemostatic stability and prevent rebleeding, the ligator was reloaded with a second nylon rope, and a second ligation was performed immediately. Third, second-layer ligation: a second nylon rope was loaded onto the ligator and deployed just above the first ligature ring to double-compress the bleeding vessel (Fig. 3C). Fourth, postoperative reinforcement: after ligation, no active bleeding was observed on the wound surface. Twenty mL of ice-cold noradrenaline saline was sprayed on the ulcer surface to further consolidate the hemostatic effect, and the endoscope was withdrawn (see Supplementary Video).

**Video 1.** 

**Figure 3.**
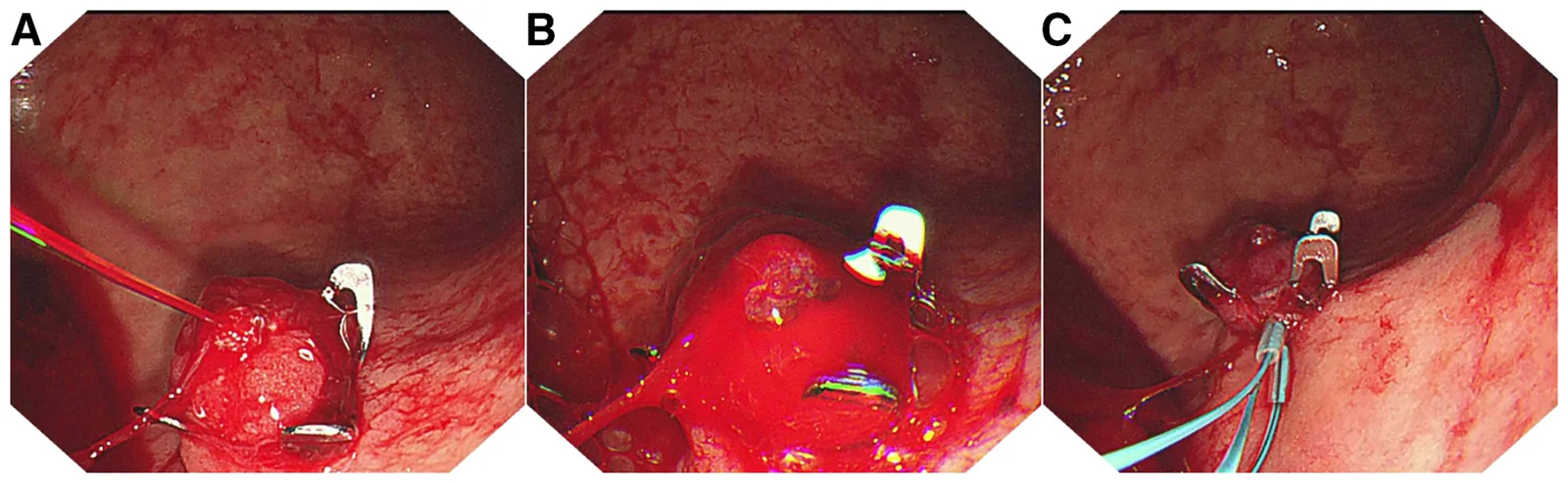
(A, B) Intermittent jet bleeding was observed at the site of the over-the-scope clip; the OTSC clip remained in place, clearly exposing the location of the vascular stump. (C) The base of the ulcer was ligated with a double-layer nylon snare, and the bleeding ceased. OTSC = over-the-scope clip.

#### 2.3.4. Postoperative management and follow-up

After returning to the ward, the patient was given nothing by mouth for 24 hours, intravenous fluid replacement and volume expansion (crystalloids + colloids), transfusion of 4 units of packed red blood cells to correct anemia, continuous infusion of esomeprazole (80 mg) for acid suppression, somatostatin to reduce GI blood flow, and nutritional support. No recurrence of hematemesis or melena was observed postoperatively, and vital signs gradually stabilized (blood pressure 110/70 mm Hg, pulse 90 beats/minute at 6 hours postoperatively; hemoglobin concentration increased to 72 g/L at 24 hours postoperatively) (Fig. 4).

**Figure 4.**
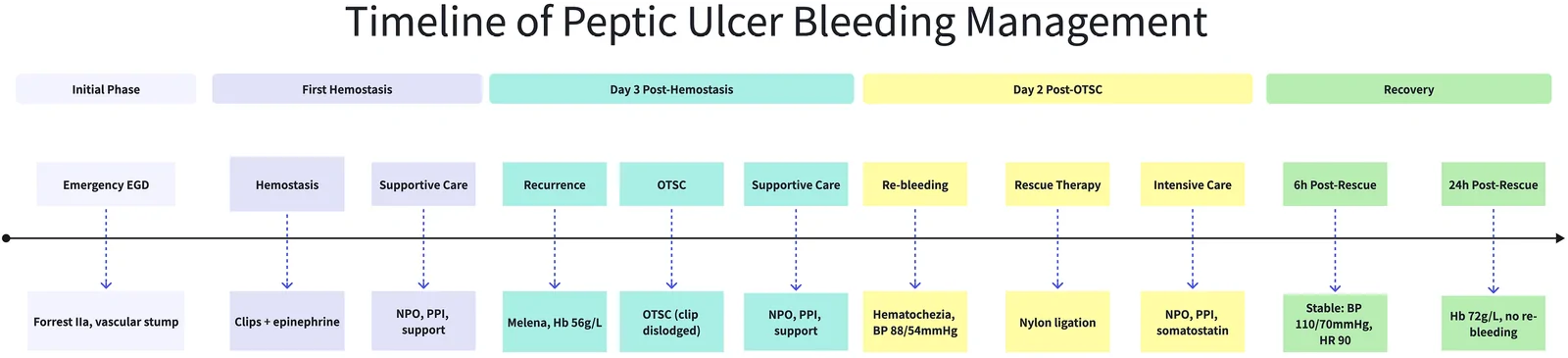
Treatment timeline of layered hemostasis for gastric ulcer bleeding. BP = blood pressure, EGD = esophagogastroduodenoscopy, Hb = hemoglobin, NPO = nil per os (nothing by mouth), OTSC = over-the-scope clip, PPI = proton pump inhibitor.

Follow-up results: gastroscopic reexamination at 2 weeks post-surgery revealed shedding of the OTSC and nylon ropes, with no active bleeding and a clean ulcer surface (Fig. 5). Routine blood tests at 2 weeks post-surgery revealed that the patient’s hemoglobin concentration had increased to 82 g/L. Gastroscopic reexamination at 8 weeks postoperatively revealed that the yellowish-white exudate on the original ulcer site at the greater curvature of the stomach had disappeared, presenting new red mucosa with slight mucosal convergence (Fig. 6). At the 4-month follow-up, the patient was asymptomatic, and the ulcer had completely healed.

**Figure 5.**
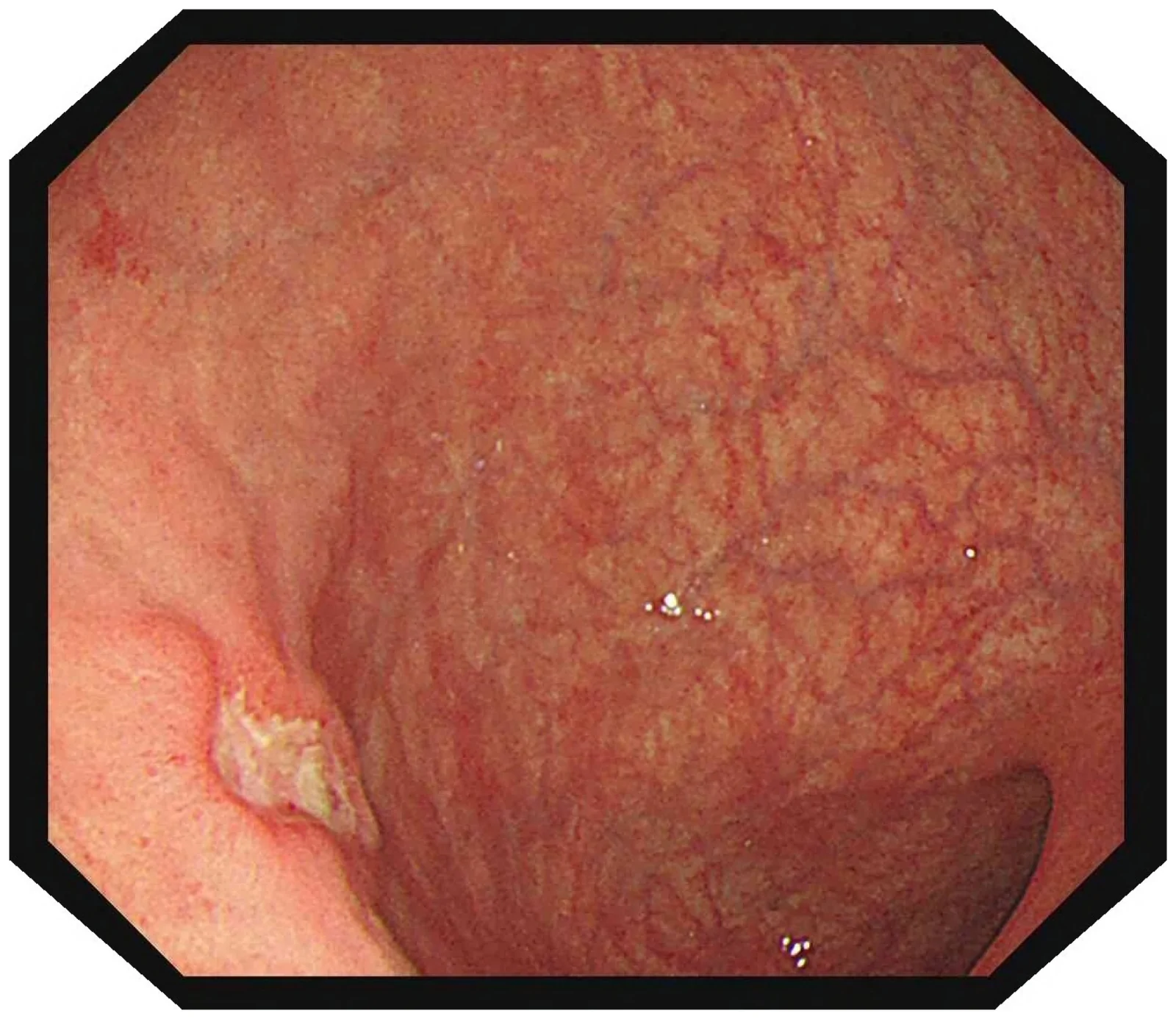
Endoscopically, the ulcer was found to be smaller than before, with the OTSC and nylon ropes shed, a clean ulcer surface, and no active bleeding. OTSC = over-the-scope clip.

**Figure 6.**
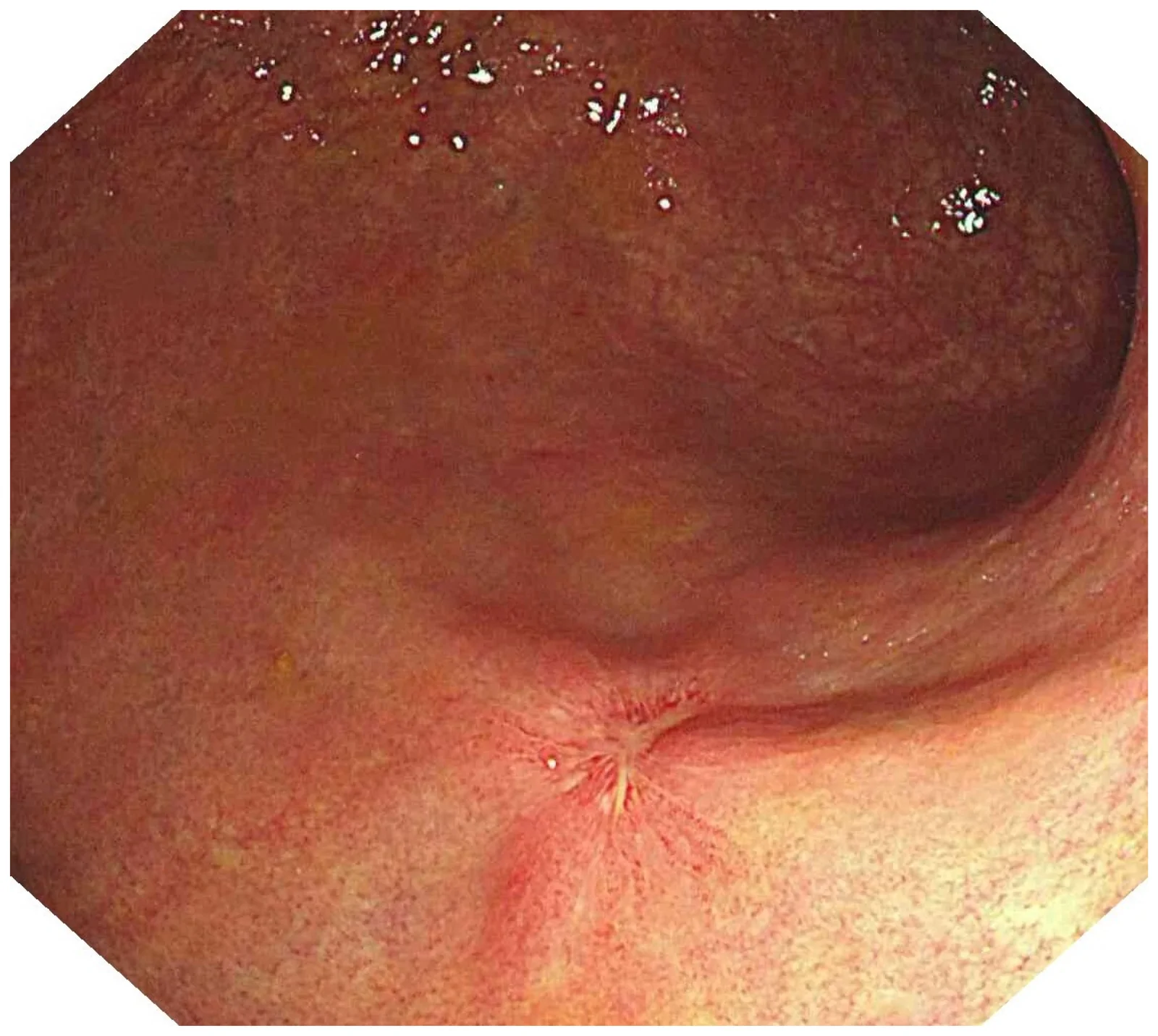
The yellowish-white exudate on the original ulcer site at the greater curvature of the stomach had disappeared, presenting new red mucosa with slight mucosal convergence.

## 3. Discussion

Gastric ulcers with bleeding are common emergencies in gastroenterology, characterized by acute onset, rapid progression, and high mortality rates. Endoscopic precise hemostasis is currently the preferred clinical treatment^[18]^ and has significantly improved the prognosis of these patients.^[19,20]^ Despite acid suppression and endoscopic treatment, 10 to 20% of patients still experience ongoing or recurrent bleeding, which remains one of the most important predictors of mortality. Therefore, improving endoscopic hemostasis methods for high-risk populations to prevent rebleeding and thereby reduce mortality is highly important. The OTSC system, a potent mechanical hemostatic tool, has been widely used to treat patients with high-risk gastric ulcer bleeding, such as those with active bleeding and exposed blood vessels, but its clinical failure rate is approximately 10 to 20%.^[11,12]^ However, experience with rescue therapy after such treatment failure is limited. In this case, jet bleeding persisted after spraying, injection hemostasis, and OTSC clipping failed, suggesting that the bleeding vessel was a submucosal arterial branch of the stomach (rather than a capillary or small vein) with high intravascular pressure. Although OTSCs are potent mechanical hemostatic tools, their sustained compression efficacy for simple mechanical clipping of arterial bleeding with a diameter > 1.5 mm is limited.^[13]^ Even if initial clipping is successful, long-term high-pressure blood flow may gradually open the tissue space at the clipped site, leading to rebleeding. The ulcer in this case was only 1.0 cm × 0.8 cm (not a large ulcer), but the bleeding vessel may have been located deep at the base of the ulcer, on the surface of the submucosa or muscularis propria, rather than the superficial mucosa. The clipping depth of an OTSC is usually limited to the mucosa and superficial submucosa.^[21]^ In addition, the patient’s gastric ulcer was located in the upper segment of the greater curvature of the gastric body, which is a relatively large area of the gastric cavity. The contractile and peristaltic activity of the gastric wall here is significantly greater than that of the angular incisure, antrum, and other sites. The high mobility of the gastric wall here leads to insufficient stability of the clipping. After OTSC clipping, the continuous activity of the gastric wall exerts repeated tensile stress on the clipped site. Even if the initial clipping position is accurate, long-term mechanical traction may cause displacement of the clipped tissue, allowing the compressed blood vessel to escape from the compression range and recreating jet bleeding. In this case, OTSC was used after “spraying + injection hemostasis” failed. The local mucosa developed edema, congestion, and even partial tissue degeneration after drug injection. The clipping efficacy of OTSCs relies on the firm clamping of “healthy, elastic mucosal tissue,” while edematous tissue is loose and has poor pressure resistance. Even if the OTSC is in place, it is difficult to achieve sustained and effective vascular compression by clamping edematous tissue, and high-pressure blood flow is prone to leakage from the tissue space or breaking through the compression site. Although the ulcer in this case was not large, active bleeding suggested that there may have been a necrotic tissue layer at the base of the ulcer.^[22]^ If the OTSC only clamped the necrotic tissue on the ulcer surface (rather than the deep normal tissue + vascular root) during clipping, the necrotic tissue would gradually slough off postoperatively, leading to reexposure of the previously covered bleeding vessel. At this time, the OTSC, which lacks anchoring to healthy tissue, cannot synchronously compress the newly exposed blood vessel as the necrotic tissue sloughs off, ultimately causing jet bleeding.

After OTSC hemostasis fails, common clinical rescue therapies include repeated endoscopic treatment, interventional therapy, and surgical treatment. Although surgical treatment can achieve complete hemostasis, it is associated with significant trauma and slow postoperative recovery and is particularly suitable for critical situations such as complicated perforation and shock. Interventional therapy requires high-precision vascular localization; may be complicated by postembolization ischemia, infection, and other complications; and has limited efficacy for peripheral vascular bleeding.^[21]^ In contrast, endoscopic nylon rope ligation achieves hemostasis through mechanical compression of the vascular wall, with simple operation, minimal tissue damage, and no need for additional special equipment. It has been reported in the literature that nylon rope ligation of the roots of large pedunculated polyps can effectively block blood supply and reduce postoperative bleeding.^[23]^ However, there have been no reports of its use for GI bleeding or as a rescue therapy after failed OTSC treatment. Based on the abovementioned therapeutic principles of nylon rope and reported cases of successful prevention of postoperative bleeding after resection of large pedunculated polyps, we selected nylon rope ligation as the rescue therapy and adopted a “double-layer” modified design to address the insufficient compression strength and easy slippage of single-layer ligation for larger blood vessels in an innovative manner.

It is important to clarify that the device used in this case was an endoscopic nylon rope ligator (Loop20-LD230, LeoMed), which is a single-layer ligator that delivers 1 nylon rope at a time. The nylon rope is loaded onto the ligator, advanced to the target site, and released to form a ligature ring. The “double-layer” hemostatic effect was achieved by loading the ligator with a nylon rope twice and performing 2 sequential ligations. After the first nylon rope was deployed at the root of the bleeding vessel, bleeding was significantly reduced. However, considering the intraoperative assessment (namely, the relatively large caliber of the visible vascular stump [approximately 0.3 cm], the severe edema and fragility of the surrounding mucosa following failed OTSC and prior injection therapy, and the strong peristaltic activity of the gastric body’s greater curvature) we were concerned that a single ligature might be prone to slippage under mechanical traction. Therefore, the ligator was reloaded with a second nylon rope, and a second ligation was performed immediately just above the first to enhance mechanical stability and ensure definitive hemostasis. This approach differs from using a predesigned double-layer device; rather, it represents an intraoperative decision to apply a second single-rope ligation based on the observed effect of the first. A single nylon rope ligation is generally adequate for small-caliber vessels (< 1 mm) or pedunculated polyps, where the mechanical compression from 1 ligature is sufficient to occlude the vascular lumen, and the risk of slippage is relatively low. However, in the present case, several factors rendered a single ligation potentially insufficient: the visible vascular stump suggested a relatively large submucosal arterial branch that would likely exceed the compressive threshold of a single rope; the surrounding mucosa was severely edematous and fragile, significantly increasing the risk of ligature slippage due to poor tissue anchoring; and the gastric body’s greater curvature is a highly mobile region with strong peristaltic activity, which may dislodge a single ligature over time through repeated mechanical traction. In contrast, 2 sequential single-rope ligations provide cumulative compressive force that exceeds the threshold for larger-caliber arterial vessels, while the second ligation acts as a mechanical safety anchor that reduces the risk of displacement caused by gastric peristalsis or edematous tissue deformation. Thus, while a single ligation may be successful in less challenging scenarios, 2 sequential ligations are specifically indicated for high-pressure arterial bleeding, significant tissue edema, or anatomically mobile locations where OTSC has already failed.

The double-layer nylon rope ligation used in this case optimized the operational logic on the basis of conventional single-layer ligation, with the core advantages reflected in 3 aspects. First, enhanced compression stability. The first layer of nylon rope was ligated at the root of the bleeding vessel to achieve basic compression, and the second layer was ligated just above it to further reduce the size of the vascular lumen through double mechanical compression, enhancing hemostatic stability and effectively compensating for the residual bleeding risk caused by inadequate OTSC clipping. Second, operational safety and controllability. No energy therapy, such as cauterization, was required during ligation, avoiding secondary damage to the edematous mucosa surrounding the ulcer and reducing the risk of perforation. Moreover, the tightening force of the nylon rope could be precisely adjusted through endoscopic operation, adapting to the tolerance of edematous tissue. Third, high operational efficiency. The entire ligation process took only 5 minutes, quickly controlling bleeding and advancing the patient’s subsequent treatment. Immediate hemostasis was achieved after the procedure, with no recurrence of hematemesis, melena, or other symptoms of active bleeding. Routine blood tests at 24 hours postoperatively showed a stable increase in hemoglobin levels compared with preoperative levels. Endoscopic reexamination at 2 weeks postoperatively showed good healing of the bleeding site with no active bleeding or ligation-related complications. The ulcer had essentially healed at 4 months of follow-up.

## 4. Conclusion

Through a detailed review and in-depth analysis of a single case of double-layer nylon rope ligation as a rescue therapy for a patient with gastric ulcer bleeding after failed OTSC, this study has important value for theoretical exploration, clinical practice, and the technical promotion of endoscopic diagnosis and the treatment of GI bleeding, providing a key reference for the diagnosis and treatment of such complex diseases. Reports on rescue therapies for peptic ulcer bleeding after failed OTSC treatment are rare, and this is the first report of endoscopic double-layer nylon rope ligation. Double-layer nylon rope ligation is simple, safe, and effective and enhances hemostatic stability through double mechanical compression. It can be used as an ideal rescue therapy for patients with gastric ulcer bleeding with a retained OTSC after failed OTSC treatment and is especially suitable for bleeding scenarios with endoscopically accessible lesions, large-diameter blood vessels, or significant tissue edema. This case provides a new practical reference for the clinical management of such high-risk bleeding. Further large-sample, multicenter retrospective or prospective studies are needed to verify its long-term efficacy and applicable scope to better guide clinical practice. As a single-center, single-case report, this study has certain limitations. First, the sample size is small, and the long-term efficacy and applicable scope of double-layer nylon rope ligation need to be verified by large-sample, multicenter retrospective or prospective studies. Second, no efficacy comparison was made with other rescue therapies (such as repeat OTSC, interventional therapy, surgical treatment, etc); thus, its advantages in terms of cost-effectiveness, operational difficulty, and other aspects cannot be clarified.

## Acknowledgments

This article specifically acknowledges Director Sun Dayong for serving as chief surgeon for the entire procedure.

## Author contributions

**Conceptualization:** Dan Wu, Da Yong Sun, Xiang Yu Wang.

**Data curation:** Dan Wu.

**Formal analysis:** Dan Wu, Hong Wei.

**Investigation:** Dan Wu.

**Methodology:** Dan Wu, Hong Wei, Xiang Yu Wang.

**Resources:** Dan Wu.

**Software:** Hong Wei, Xiang Yu Wang.

**Supervision:** Da Yong Sun.

**Validation:** Dan Wu, Da Yong Sun.

**Writing – original draft:** Dan Wu, Hong Wei, Xiang Yu Wang.

**Writing – review & editing:** Dan Wu, Hong Wei, Xiang Yu Wang.

## References

[1] Gralnek IM Barkun AN Bardou M. Management of acute bleeding from a peptic ulcer. N Engl J Med. 2008;359:928–37. 10.1056/NEJMra0706113 18753649

[2] van Leerdam ME. Epidemiology of acute upper gastrointestinal bleeding. Best Pract Res Clin Gastroenterol. 2008;22:209–24. 10.1016/j.bpg.2007.10.011 18346679

[3] Gralnek IM Dumonceau JM Kuipers EJ. Diagnosis and management of nonvariceal upper gastrointestinal hemorrhage: European Society of Gastrointestinal Endoscopy (ESGE) Guideline. Endoscopy. 2015;47:a1–46. 10.1055/s-0034-1393172 26417980

[4] Barkun AN Bardou M Kuipers EJ. International consensus recommendations on the management of patients with nonvariceal upper gastrointestinal bleeding. Ann Intern Med. 2010;152:101–13. 10.7326/0003-4819-152-2-201001190-00009 20083829

[5] Götz M Anders M Biecker E. [S2k guideline gastrointestinal bleeding - guideline of the German society of gastroenterology DGVS]. Z Gastroenterol. 2017;55:883–936. 10.1055/s-0043-116856 29186643

[6] Laine L Yang H Chang SC Datto C. Trends for incidence of hospitalization and death due to GI complications in the United States from 2001 to 2009. Am J Gastroenterol. 2012;107:1190–5; quiz 1196. 10.1038/ajg.2012.168 22688850

[7] Van Leerdam ME Vreeburg EM Rauws EAJ. Acute upper GI bleeding: did anything change? Time trend analysis of incidence and outcome of acute upper GI bleeding between 1993/1994 and 2000. Am J Gastroenterol. 2003;98:1494–9. 10.1111/j.1572-0241.2003.07517.x 12873568

[8] Mullady DK Wang AY Waschke KA. AGA clinical practice update on endoscopic therapies for non-variceal upper gastrointestinal bleeding: expert review. Gastroenterology. 2020;159:1120–8. 10.1053/j.gastro.2020.05.095 32574620

[9] Chan S Pittayanon R Wang HP. Use of over-the-scope clip (OTSC) versus standard therapy for the prevention of rebleeding in large peptic ulcers (size ≥1.5 cm): an open-labelled, multicentre international randomised controlled trial. Gut. 2023;72:638–43. 10.1136/gutjnl-2022-327007 PMC10086285 36307177

[10] Laine L Barkun A Saltzman J. ACG clinical guideline: Upper gastrointestinal and ulcer bleeding. Am J Gastroenterol. 2021;116:899–917. 10.14309/ajg.0000000000001245 33929377

[11] Schmidt A Gölder S Goetz M. Over-the-scope clips are more effective than standard endoscopic therapy for patients with recurrent bleeding of peptic ulcers. Gastroenterology. 2018;155:674–86.e6. 10.1053/j.gastro.2018.05.037 29803838

[12] Brandler J Baruah A Zeb M. Efficacy of over-the-scope clips in management of high-risk gastrointestinal bleeding. Clin Gastroenterol Hepatol. 2018;16:690–6.e1. 10.1016/j.cgh.2017.07.020 28756055

[13] Asokkumar R Sanchez-Yague A Soetikno R. Incomplete hemostasis of high-risk adverse outcome bleeding lesions after placement of the over-the-scope clip: causes and solutions. VideoGIE. 2018;3:155–6. 10.1016/j.vgie.2018.02.005 PMC6004426 29916458

[14] Laine L Jensen DM. Management of patients with ulcer bleeding. Am J Gastroenterol. 2012;107:345–60; quiz 361. 10.1038/ajg.2011.480 22310222

[15] Tarasconi A Luca Baiocchi G Pattonieri V. Transcatheter arterial embolization versus surgery for refractory non-variceal upper gastrointestinal bleeding: a meta-analysis. World J Emerg Surg. 2019;14:3. eCollection 2019 10.1186/s13017-019-0223-8 PMC6359767 30733822

[16] Roy A Kim M Hawes R Varadarajulu S. The clinical and cost implications of failed endoscopic hemostasis in gastroduodenal ulcer bleeding. United Eur Gastroenterol J. 2017;5:359–64. 10.1177/2050640616663570 PMC5415211 28507747

[17] Xie YX Zheng YL Tang Q Wang DM. Case Report: Endoscopic resection of giant colonic pedunculated polyps in patients with AIDS using a novel nylon rope and titanium clip technique. Front Oncol. 2025;15:1566005. 10.3389/fonc.2025.1566005 PMC12116440 40438680

[18] Ahn D-W Soo Park Y Hyub Lee S. Clinical outcome of acute nonvariceal upper gastrointestinal bleeding after hours: the role of urgent endoscopy. Korean J Intern Med. 2016;31:470–8. 10.3904/kjim.2014.099 PMC4855084 27048253

[19] Cook DJ Guyatt GH Salena BJ Laine LA. Endoscopic therapy for acute nonvariceal upper gastrointestinal hemorrhage: a meta-analysis. Gastroenterology. 1992;102:139–48. 10.1016/0016-5085(92)91793-4 1530782

[20] Soriani P Biancheri P Francesco Bonura G. Over-the-scope clip as first-line treatment of peptic ulcer bleeding: a multicenter randomized controlled trial (TOP Study). Endoscopy. 2024;56:665–73. 10.1055/a-2303-4824 38599622

[21] Wedi E Renteln D von Gonzalez S. Use of the over-the-scope-clip (OTSC) in non-variceal upper gastrointestinal bleeding in patients with severe cardiovascular comorbidities: a retrospective study. Endosc Int Open. 2017;5:E875–82. 10.1055/s-0043-105496 PMC5595576 28924593

[22] Zhang MM Ji L Lan T. Design of magnamosis system for endoscopic tissue clamping. Chin J Med Instrumentation. 2021;45:612–5. (1671-7104(2021)06-0612-04) 10.3969/j.issn.1671-7104.2021.06.006 34862772

[23] Chou K-C Yen H-H. Combined endoclip and endoloop treatment for delayed postpolypectomy hemorrhage. Gastrointest Endosc. 2010;72:218–9. 10.1016/j.gie.2009.11.001 20226454

